# Supplementary material for: Proteomics of Bacterial and Mouse Extracellular Vesicles Released in the Gastrointestinal Tracts of Nutrient-Stressed Animals Reveals an Interplay Between Microbial Serine Proteases and Mammalian Serine Protease Inhibitors
Source: Int J Mol Sci. 2025 Apr 25;26(9):4080. doi: 10.3390/ijms26094080 (PMC12071298; doi:10.3390/ijms26094080)
Supplement: Supplementary file 1 [file ijms-26-04080-s001.zip › ijms-3569233-supplementary.pdf]

Table S3 Polysaccharide Utilization Units

Accession	Description	# PSMs	Ratio: (Fasted) / (Non-Fasted)	Literature derived	Substrate
Q8AAZ5	SusC homolog BT_0317	34	1.676	**PUL 6**	Mucin O-glycans (N-acetyllactosamine, adult and suckling mouse) [50]
Q8AAZ4	SusD homolog BT_0318	5	1.2	BT0317-	
Q8AAZ3	SusD homolog BT_0319	1	1.207	BT319	
Q8AAM5	SusC homolog BT_0439	13	1.508	**PUL 8**	Host/residual dietary glycans (unknown type) [50]
Q8AAM4	SusD homolog BT_0440	2	0.647	BT0433-	
Q8AAM3	Putative chitobiase BT_0441	1	0.984	BT0445	
Q8AAL5	Uncharacterized protein BT_0449	2	0.561	**PUL 9**	Host glycans (unknown type, PMG, adult and suckling mice) [50]
Q8AAL3	SusD homolog BT_0451	5	0.904	BT0446-	
Q8AAL2	SusC homolog BT_0452	26	1.325	BT0461	
Q8AAK9	Sialidase (Neuraminidase) BT_0455	1	1.049		
Q8AAK5	Beta-hexosaminidaseBT_0459	1	1.322		
Q8A8Y5	Alpha-1,2-mannosidase family protein BT_1032	1	0.887	**PUL 14**	Mucin O-glycans [50]
Q8A8Y4	1,4-beta-mannosyl-N-acetylglucosamine phosphorylase BT_1033	7	1.64	BT1032-	Complex N-glycan (3/5) [71]
Q8A8Y1	Coagulation factor 5/8 type BT_1036	1	0.739	BT1053	
Q8A8Y0	Concanavalin A-like lectin/glucanase BT_1037	9	0.871		
Q8A8X9	Putative secreted endoglycosidase BT_1038	8	1.074		
Q8A8X8	SusD homolog BT_1039	14	1.405		
Q8A8X7	SusC homolog BT_1040 PE=3 SV=1	26	1.937		
Q8A8X5	SusC homolog BT_1042	38	1.156		
Q8A8X4	SusD homolog BT_1043	9	1.102		
Q8A8X3	Putative secreted endoglycosidase, GH family 18 BT_1044	7	1.001		
Q8A8X2	Concanavalin A-like lectin/glucanase BT_1045	13	0.973		
Q8A8X1	SusC homolog BT_1046	15	1.92		
Q8A8X0	SusD homolog BT_1047	3	0.91		
Q8A8W9	Putative secreted endoglycosidase BT_1048	2	1.065		
Q8A8W8	Putative patatin-like protein BT_1049	3	1.366		
Q8A7T4	SusC homolog BT_1440	37	1.448	**PUL 17**	ND
		30	1.241	BT1439-	
Q8A7T5	SusD homolog BT_1439			BT1440	
Q8A742	SusC homolog BT_1683	33	1.335	**PUL 21**	Rhamnogalacturonan II [72]
		5	0.959	BT1682-	Rhamnogalacturonan II (2/3) [73]
Q8A743	SusD homolog BT_1682			BT1683	
Q8A5H1	SusC homolog BT_2268	115	1.31	**PUL 30**	ND
Q8A5H0	SusD homolog BT_2269	26	0.864		

				BT2268-BT2269	
Q8A4N9	SusC homolog BT_2560	17	1.801	**PUL 35**	Mucin O-glycans (N-acetyllactosamine disaccharide) [50]
Q8A4P0	SusD homolog BT_2559	7	1.414	BT2559-2562	
Q8A4H3	SusC homolog BT_2626	19	1.543	**PUL 36**	Alpha-mannan, host N-glycans [50]
Q8A4H4	SusD homolog BT_2625	4	0.723	BT2615-	
Q8A4H0	Putative alpha-1,2-mannosidase BT_2629	2	0.739	BT2633	
Q8A3Z9	SusC homolog BT_2805	14	1.567	**PUL 37**	host glycans (unknown type, PMG, adult and suckling mice) [50] - ribose-containing nutrients [74]
		2	1.024	BT2802-	
Q8A3Z8	SusD homolog BT_2806			BT2809	
Q8A1G1	TonB-dependent receptor SusC BT_3702	41	1.453	**PUL 66**	Starches [50]
Q8A1G2	Starch-binding protein SusD BT_3701	27	1.329	BT3698-	
G8JZS4	Glucan 1,4-alpha-glucosidase SusB BT_3703	13	0.855	BT3705	
Q8A1G3	Alpha-amylase SusG BT_3698	10	0.65		
G8JZT0	Outer membrane protein SusE BT_3700	7	0.755		
G8JZS6	Outer membrane protein SusF BT_3699	5	1.015		
Q8A0I3	SusD homolog BT_4038	6	0.853	**PUL 73**	Mucin O-glycans [50]
Q8A0I2	SusC homolog BT_4039	21	1.387	BT4038-BT4040	
Q8A069	Beta-galactosidase I BT_4152	1	1.029	**PUL 77**	Rhamnogalacturonan I [72]
Q8A057	SusC homolog BT_4164	13	1.218	BT4145-	
Q8A056	SusD homolog BT_4165	2	0.819	BT4183	
Q8A055	Putative lipoprotein BT_4166	4	1.075		
Q8A053	SusC homolog BT_4168	15	1.328		
Q8A054	Uncharacterized protein BT_4167	4	0.767		
Q8A052	SusD homolog BT_4169	6	0.666		
Q89ZX8	Glycosyl hydrolase family 109 protein 1 BT_4243	8	0.768	**PUL 78**	Mucin O-glycans [50]
Q89ZX7	Uncharacterized protein BT_4244	13	0.608	BT4240-	
Q89ZX4	SusC homolog BT_4247	58	1.282	BT4250	
Q89ZX5	SusD homolog BT_4246	28	0.848		
Q89ZX6	Uncharacterized protein BT_4245	5	0.78		
Q89ZS8	SusC homolog BT_4298	54	1.451	**PUL 80**	Host glycans (unknown type, likely mucin O-glycans) [50]
Q89ZS9	SusD homolog BT_4297	16	1.031	BT4294-	
Q89ZT1	Putative chitobiase BT_4295	13	0.757	BT4300	
Q89ZT0	MscS mechanosensitive ion channel BT_4296	8	1.05		

Q89ZS7	Putative cell surface protein BT_4299	14	0.972		
Q89ZM0	SusC homolog BT_4357	23	1.865	**PUL 81**	Mucin O-glycans [50]
		11	1.546	BT4355-	
Q89ZL9	SusD homolog BT_4358			BT4359	
Q89ZH3	SusC homolog BT_4404	13	1.72	**PUL 82**	Mucin O-glycans [50]
Q89ZH1	Putative secreted endoglycosidase BT_4406	2	0.71	BT4402-	Complex N-glycan (5/5) [71]
				BT4407	
Q89YR4	Beta-galactosidase BT_4667	1	2.73	**PUL 86**	Pectic galactan [50] [72]
Q89YR2	Uncharacterized protein BT_4669	3	0.865	BT4667-	
Q89YR1	SusD homolog BT_4670	14	1.067	BT4673	
Q89YR0	SusC homolog BT_4671	33	1.311		
Q89YM4	SusC homolog BT_4707	14	1.362	**PUL 87**	ND
Q89YM3	SusD homolog BT_4708	6	0.746	BT4705-	
Q89YM2	Glycoside hydrolase family 18 BT_4709	1	0.635	4714	
Q89YM1	Concanavalin A-like lectin/glucanaseBT_4710	3	1.067		
Q89YM0	Uncharacterized protein BT_4711	1	0.67		
Q8A7A6	SusC homolog BT_1619	16	1.648	**PUL19**<br>BT1617-<br>BT1622	Host glycans (unknown type, adult and suckling mouse) [50]
Q8A1I1	SusC homolog BT_3680	12	1.575	**PUL 65**	Arabinogalactan [72]
Q8A1I0	SusD homolog BT_3681	7	1.004	BT3674-	
Q8A1H6	Beta-glucanase BT_3685	2	0.659	BT3687	
Q8A0R9	SusC homolog BT_3952	5	1.754	**PUL 70**<br>BT3951-<br>BT3956	ND
Q8A1Q5	SusC homolog BT_3604	5	1.73	**PUL62**	ND
Q8A1Q6	SusD homolog BT_3603	1	0.859	BT3581-	
				BT3609	
Q8A1Y9	SusC homolog BT_3519	8	1.564	**PUL60**	ND
Q8A1Y8	SusD homolog BT_3520	2	0.733	BT3517-	
Q8A1Y7	Alpha-1,6-mannanase BT_3521	1	0.29	BT3532	
Q8A214	SusC homolog BT_3494	6	1.443	**PUL59**	Host/residual dietary glycans (unknown type) [50]
				BT3461-	
Q8A213	SusD homolog BT_3495	4	1.218	BT3507	

Q8A202	SusD homolog BT_3506	3	1.01		
Q8A2N4	SusC homolog BT_3271	4	1.208	**PUL53** BT3269-BT3276	ND
Q8A1B6	SusC homolog BT_3750	3	1.796	**PUL67** BT3748-BT3754	Mucin O-glycans [50]
Q8A2Z9	SusC homolog BT_3156	4	1.262	**PUL50** BT3155-BT3159	ND
Q8AAI1<br>Q8AAI0	SusC homolog BT_0483<br>SusD homolog BT_0484	4<br>4	2.126<br>1.184	**PUL10** BT3183-BT3184	ND
Q8A365	SusC homolog BT_3090	6	1.547	**PUL48** BT3086-BT3091	ND
Q8A2J5	SusC homolog BT_3310	3	1.7	**PUL56** BT3309-BT3314	1,6-beta-glucan [50,75]
Q8A3N4	SusC homolog BT_2920	3	1.486	**PUL42** BT2911-BT2923	ND
Q8A231<br>Q8A232	SusC homolog BT_3475<br>SusD homolog BT_3474	6<br>1	1.553<br>1.108	**PUL48** BT3461-BT3507	host/residual dietary glycans (unknown type) [50]
Q8A3Y6<br>Q8A3Y4	SusC homolog BT_2818<br>SusC homolog BT_2820	3<br>3	1.379<br>1.826	**PUL38** BT2818-BT2826	mucin O-glycans [50]
Q8A3E2	SusC homolog BT_3012	2	2.048	**PUL45** BT3010-BT3017	host glycans (unknown type, PMG phase 2) [50]
Q89YK7	SusC homolog BT_4724	2	1.95	**PUL88**	ND

				BT4722-BT4728	
Q89YU7	SusC homolog BT_4634	3	2.861	**PUL84** BT4631-BT4636	mucin O-glycans (N-acetyllactosamine disaccharide) [50]
Q8A9E8	SusC homolog BT_0867	2	1.702	**PUL12** BT0865-BT0867	mucin O-glycans (N-acetyllactosamine, adult and suckling mouse) [50]
Q8A9E9	SusD homolog BT_0866	3	1.188		
Q8A3U5	SusC homolog BT_2859	2	1.557	**PUL39** BT2851-BT2860	ND
